# Purinergic Signaling in Bone

**DOI:** 10.1155/2013/673684

**Published:** 2013-05-16

**Authors:** Niklas Rye Jørgensen, Elena Adinolfi, Isabel Orriss, Peter Schwarz

**Affiliations:** ^1^Research Center for Ageing and Osteoporosis, Departments of Diagnostics and Medicine, Copenhagen University Hospital Glostrup, 2600 Glostrup, Denmark; ^2^Department of Morphology, Surgery and Experimental Medicine, Section of General Pathology, University of Ferrara, 44121 Ferrara, Italy; ^3^Bone Biology Laboratory, Department of Cell and Developmental Biology, University College London, London WC1E 6BT, UK; ^4^Faculty of Health Sciences, University of Copenhagen, 2200 Copenhagen, Denmark

In recent years, it has become apparent that extracellular nucleotides, signalling via P2 receptors, play an important role in the regulation of bone turnover. Furthermore, purinergic signalling has been associated in the pathophysiology of several bone and cartilage diseases, including osteoarthritis, rheumatoid arthritis, and osteoporosis and might also be implicated in the deleterious skeletal effects of cancer and on bone pain. Widespread expression of multiple P2 receptor subtypes by bone and cartilage cells has now been reported, and the functional effects of receptor activation are being determined. Of all of the P2 receptors expressed, it is the P2X7 receptor which has emerged as being central in the pathogenesis of several skeletal conditions, though also a number of other P2Y and P2X receptors have important roles in regulation of bone turnover.

There are many studies, using human and animal models, which have described the pivotal role of the P2X7 receptor in rheumatoid arthritis, a complex, multifactorial inflammatory disease with no current successful treatment. The review entitled “*Modulating P2X7 receptor signaling during rheumatoid arthritis: new therapeutic approaches for bisphosphonates*” by A. Baroja-Mazo and P. Pelegrin included in this special issue summarises some of this research. In particular, it focuses on the therapeutic potential of P2X7 receptor antagonists, both alone and in combination with bisphosphonates, as a treatment for rheumatoid arthritis.

Recent work has shown that the P2X7 receptor is also important in bone-related conditions, particularly osteoporosis. A number of clinical studies have associated single-nucleotide polymorphisms (SNPs) in the P2X7 receptor gene with increased fracture risk, low bone mineral density, and increased bone loss in humans. The importance of genetic variation within the P2X7 receptor in relation to bone mass and strength is addressed by two original research articles by S. Syberg et al. in this special issue. Firstly, the paper entitled “*Association between P2X7 receptor polymorphisms and bone status in mice*” investigates the effects of one SNP (P451L) using an *in vivo *animal model. The second article entitled “*Genetic background strongly influences the phenotype of P2X7 receptor knockout mice*” provides a detailed analysis of the differences of bone phenotype between two different strains of P2X7 receptor knockout. Combinedly these research papers highlight the importance of genetic background when looking at the functional effects of the P2X7 receptor and suggest that when mouse models are used to test the efficacy of P2X7 receptor agonists and antagonists it is taken into account. Also they demonstrate the role of the P2X7 receptor in regulation of bone mass.

Bone is both a site of primary tumor formation and metastatic spread of high incidence neoplasias such as breast and prostate cancers. To date, the efficacy of therapies intended to reduce bone alterations and related pain in cancer is limited. In this special issue, two papers point out to P2X receptors as possible targets for the treatment of bone cancer and associated pain. E. Adinolfi et al. cover recent findings linking P2X7 receptor and bone biology with a focus on P2X7-mediated osteoblast proliferation and osteoclast differentiation. The authors report evidence on the role of the P2X7 receptor as an oncogene implicated in cancer growth, neovascularization, and metastatic dissemination. Their paper relies on data reported by the recent literature and examination of Affymetrix-based expression databases. Examined tumors include osteosarcoma, neuroblastoma, multiple myeloma, and breast and prostate cancers.

The review from S. Falk et al. is centered on the role of purinergic nociception in cancer-induced bone pain. In particular, the authors report evidence in favor of P2X3 involvement in bone-related pain both at the peripheral and central levels concentrating on studies conducted in different rodent models of cancer-induced pain states. The authors also report on the role of other P2X and P2Y receptors in the complex network of cells involved in bone pain development, with a critical perspective taking into account all the possible problems linked to model representative potential. *“P2X7 receptor function in bone-related cancer” and “The role of purinergic receptors in cancer-induced bone pain”* both take into consideration the role of the natural agonist of P2X receptors, extracellular ATP, in cancer. Indeed, several publications reported the presence of high levels of ATP in cancer microenvironment, and a role for this nucleotide in increasing the host immune response was suggested. Data summarized in the reviews by E. Adinolfi et al. and S. Falk et al. suggest a mechanism by which extracellular ATP released from tumor cells, through the P2X7 receptor, might affect osteoblast growth and osteoclast activity, while it might induce pain through the P2X3 receptor.

Increasing evidence supports the role of purinergic signaling through P2 purinergic receptors in regulating normal bone turnover, but also seems to play a role in the pathophysiology of a range of bone diseases including postmenopausal osteoporosis, immune-mediated bone loss, and cancer-induced bone disease as well as in bone-pain.

Thus, accumulating evidence provide us with a range of new therapeutic targets to treat the above-mentioned diseases, for some of which efficacious treatment options are not currently available. In terms of therapeutic strategy for cancer-induced bone disease, one could speculate that treatment with a cocktail of drugs contemporarily targeting multiple P2X receptor could prove efficacious both in reducing cancer growth, dissemination and pain sensation, while the widespread expression of P2 receptors on the different types of bone cells could prove to be a novel target for the regulation of bone formation and resorption in both postmenopausal osteoporosis and osteoporosis related to other diseases.

It will certainly be interesting to follow the continued progress in the field of purinergic signaling in bone in the future, and the growing acceptance of this concept is supported by the recent funding by the European Commission's 7th Framework Programme of the ATPBone project and a dedicated session at the European Calcified Tissue Society 2012 conference to “ATP and bone,” putting this area of research concept on the scientific map.

*Niklas Rye Jørgensen* *Niklas Rye Jørgensen* *Elena Adinolfi* *Elena Adinolfi* *Isabel Orriss* *Isabel Orriss* *Peter Schwarz* *Peter Schwarz*

